# Retraction Note: A newly isolated strain of *Halomonas* sp. (HA1) exerts anticancer potential via induction of apoptosis and G_2_/M arrest in hepatocellular carcinoma (HepG2) cell line

**DOI:** 10.1038/s41598-024-73028-0

**Published:** 2024-09-30

**Authors:** Islam M. El-Garawani, Sabha M. El-Sabbagh, Nasser H. Abbas, Hany S. Ahmed, Omaima A. Eissa, Doaa M. Abo-Atya, Shaden A. M. Khalifa, Hesham R. El-Seedi

**Affiliations:** 1 Department of Zoology, Faculty of Science, Menoufia University, Menoufia, 32511 Egypt; 2 Department of Botany and Microbiology, Faculty of Science, Menoufia University, Menoufia, 32511 Egypt; 3 Department of Molecular BiologyGenetic Engineering and Biotechnology Research Institute, University of Sadat City, Sadat City, 32958 Egypt; 4 Department of Chemistry, Faculty of Science, Menoufia University, Menoufia, 32511 Egypt; 5 Department of Molecular Biosciences, The Wenner-Gren Institute, Stockholm University, 10691 Stockholm, Sweden; 6 Pharmacognosy Group, Department of Pharmaceutical Biosciences, Uppsala University, Biomedical Centre, 75 123 Uppsala, Sweden; 7 International Research Center for Food Nutrition and Safety, Jiangsu University, Zhenjiang, 212013 China

Retraction of: *Scientific Reports*10.1038/s41598-020-70945-8, published online 21 August 2020

The Editors have retracted this Article. After publication, concerns were raised regarding the data presented in Figs. 4 and 10. Specifically,

- A portion of the micrograph shown in Fig. 4, *Control* appears to overlap with another portion of the same panel;
- A portion of the micrograph shown in Fig. 4, *DMSO* appears to overlap with another portion of the same panel;
- A portion of the micrograph shown in Fig. 4, *H. HA1 40 μg/mL* appears to overlap with another portion of the same panel;
- Two portions of the micrograph shown in Fig. 10, *H. HA1 20 μg/mL*, when rotated and re-scaled, appear to overlap with two portions of Fig. 5, *1 day TBX* in [1]. The two panels represent cells taken from animals which were subject to different conditions and treatments.

The Authors provided some of the original data, but this was insufficient to address the concerns raised. The Editors therefore no longer have confidence in the research presented in this work.

Sabha M. El-Sabbagh, Nasser H. Abbas, Hany S. Ahmed and Doaa M. Abo-Atya do not agree to this retraction. Islam M. El-Garawani did not explicitly say whether they agree or disagree to this retraction. Omaima A. Eissa, Shaden A. M. Khalifa, and Hesham R. El-Seedi did not respond to correspondence from the Editor about this retraction.
